# Amphiphilic branched polymer-nitroxides conjugate as a nanoscale agent for potential magnetic resonance imaging of multiple objects in vivo

**DOI:** 10.1186/s12951-021-00951-z

**Published:** 2021-07-09

**Authors:** Xiaoming Wang, Shiwei Guo, Zhiqian Li, Qiang Luo, Yan Dai, Hu Zhang, Yun Ye, Qiyong Gong, Kui Luo

**Affiliations:** 1 Huaxi MR Research Center (HMRRC), Department of Radiology, National Clinical Research Center for Geriatrics, Frontiers Science Center for Disease-Related Molecular Network, State Key Laboratory of Biotherapy, West China Hospital, Sichuan University, 610041 Chengdu, China; 2 Functional and Molecular Imaging Key Laboratory of Sichuan Province, Research Unit of Psychoradiology, Chinese Academy of Medical Sciences, 610041 Chengdu, China; 3 Department of Radiology, Chongqing General Hospital, University of Chinese Academy of Sciences (UCAS), No. 104 Pipashan Main Street, Yuzhong District, 400014 Chongqing, China; 4 Department of Pharmacy of the Affiliated Hospital of Southwest Medical University, Southwest Medical University, Sichuan Province 646000 Luzhou, People’s Republic of China; 5 Nuclear Medicine and Molecular Imaging Key Laboratory of Sichuan Province, 646000 Luzhou, People’s Republic of China; 6 Amgen Bioprocessing Centre, Keck Graduate Institute Claremont, 91711 Claremont, CA USA

**Keywords:** Metal-free contrast agents, Nitroxides, Magnetic resonance imaging, Polymers

## Abstract

**Background:**

In order to address the potential toxicity of metal-based magnetic resonance imaging (MRI) contrast agents (CAs), a concept of non-metallic MRI CAs has emerged. Currently, paramagnetic nitroxides (such as (2,2,5,5-tetramethylpyrrolidine-1-oxyl, PROXYL), (2,2,6,6-tetramethylpiperidine-1-oxide, TEMPO), etc.) are being extensively studied because their good stability and imaging mechanism are similar to metal-based contrast agents (such as Gd^3+^ chelate-based clinical CAs). However, a lower relaxivity and rapid in vivo metabolism of nitroxides remain to be addressed. Previous studies have demonstrated that the construction of macromolecular nitroxides contrast agents (mORCAs) is a promising solution through macromolecularization of nitroxides (i.e., use of large molecules to carry nitroxides). Macromolecular effects not only increase the stability of nitroxides by limiting their exposure to reductive substances in the body, but also improve the overall ^1^H water relaxation by increasing the concentration of nitroxides and slowing the molecular rotation speed.

**Results:**

Branched pDHPMA-mPEG-Ppa-PROXYL with a high molecular weight (MW = 160 kDa) and a nitroxides content (0.059 mmol/g) can form a nanoscale (~ 28 nm) self-assembled aggregate in a water environment and hydrophobic PROXYL can be protected by a hydrophilic outer layer to obtain strong reduction resistance in vivo. Compared with a small molecular CA (3-Carboxy-PROXYL (3-CP)), Branched pDHPMA-mPEG-Ppa-PROXYL displays three prominent features: (1) its longitudinal relaxivity (0.50 mM^− 1^ s^− 1^) is about three times that of 3-CP (0.17 mM^− 1^ s^− 1^); (2) the blood retention time of nitroxides is significantly increased from a few minutes of 3-CP to 6 h; (3) it provides long-term and significant enhancement in MR imaging of the tumor, liver, kidney and cardiovascular system (heart and aortaventralis), and this is the first report on nitroxides-based MRI CAs for imaging the cardiovascular system.

**Conclusions:**

As a safe and efficient candidate metal-free magnetic resonance contrast agent, Branched pDHPMA-mPEG-Ppa-PROXYL is expected to be used not only in imaging the tumor, liver and kidney, but also the cardiovascular system, which expands the application scope of these CAs.

**Graphical abstract:**

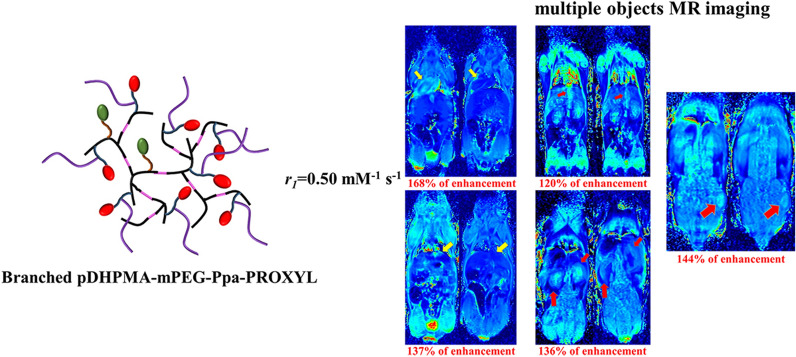

**Supplementary Information:**

The online version contains supplementary material available at 10.1186/s12951-021-00951-z.

## Introduction

Magnetic resonance imaging (MRI) is a very effective medical imaging technology which has been widely used in the clinical diagnosis of many diseases [1–3]. However, conventional MRI examinations often have insufficient image contrast, so a higher amount of MRI contrast agents need to be applied to patients to obtain clearer and more accurate diagnostic results [4–7]. At present, metal-based MRI contrast agents (CAs) are commonly used clinically, including paramagnetic Gd^3+^ chelates (T1, positive contrast agents) and superparamagnetic Fe_3_O_4_ nanoparticles (T2, negative contrast agents) [8–11]. Unfortunately, both of them have safety risks. [12–14]. Therefore, it has become a research hotspot to address the toxicity of metal-based MRI CAs.

In this process, a concept of non-metallic MRI CAs has emerged. Currently, paramagnetic nitrogen oxide radicals (nitroxides) (such as PROXYL, TEMPO, etc.) are being extensively studied because of their good stability and a similar imaging mechanism to metal-based contrast agents (such as Gd^3+^ chelate-based clinical CAs) [15–19]. However, there are some inherent obstacles of nitroxides that need to be overcome to use as clinical MRI CAs. First, the unpaired electrons (only one) of nitroxides are less than those of Gd(III) ions (seven), so the T1 relaxation efficiency (*r*_*1*_) of nitroxides are significantly lower than that of contrast agents based on Gd(III) ions. In addition, due to the reducible sensitivity, nitroxides will be quickly converted into non-relaxing nitrohydroxyl compounds by the reductive substances in the body [20–24], resulting in loss of paramagnetism, which leads to their short half-life and an insufficient window time for MR imaging. Previous studies [25–29] have demonstrated that the construction of macromolecular nitroxides-based contrast agents (mORCAs) is a promising solution *via* macromolecularization of nitroxides, or use of large molecules to carry nitroxides. The macromolecular effect not only enhances the stability of nitroxides by limiting their exposure to reductive substances in the body, but also improves the overall ^1^H water relaxation by increasing the concentration of nitroxides and slowing the molecular rotation speed [27, 30–33]. However, these mORCAs have not obtained ideal results. They mainly face two challenges to be addressed: (1) relaxivities and in vivo imaging durations need to be further improved; (2) biosafety of macromolecular materials [34]. In the early stage, we used linear and cross-linked biodegradable PEGylated polyester to construct two novel mORCAs which have made unprecedented achievements in solving the above two problems, and the cross-linked one was the most outstanding [35]. Although the above studies have not completely solved the problems faced by mORCAs, it has injected great expectation and hope into the research field. Therefore, it is necessary to develop more macromolecular carrier materials for constructing more mORCAs in order to develop efficacious metal-free MRI CAs.

Among macromolecular carriers, biodegradable poly[*N*-(1,3-dihydroxypropyl) methacrylamide] (DHPMA copolymers) are favored due to their great structure controllability, diversified functions, long-term blood circulation, excellent water solubility, non-immunogenicity and good biosafety [36–41]. These copolymers have been successfully employed for the development of metal-based macromolecular CAs (mCAs) [30, 42, 43]. Therefore, it is expected that the combination of small molecular nitroxide radicals with biodegradable DHPMA copolymers could result in safe and efficient metal-free MRI mCAs. These copolymers help nitroxides to improve the relaxivity in vitro and accumulation of nitroxides in some tissues and organs in vivo, thereby generating a multiplication effect to achieve improvement in the imaging contrast. Meanwhile, these DHPMA copolymers have been demonstrated to display low side effects [44].

**Fig. 1:**
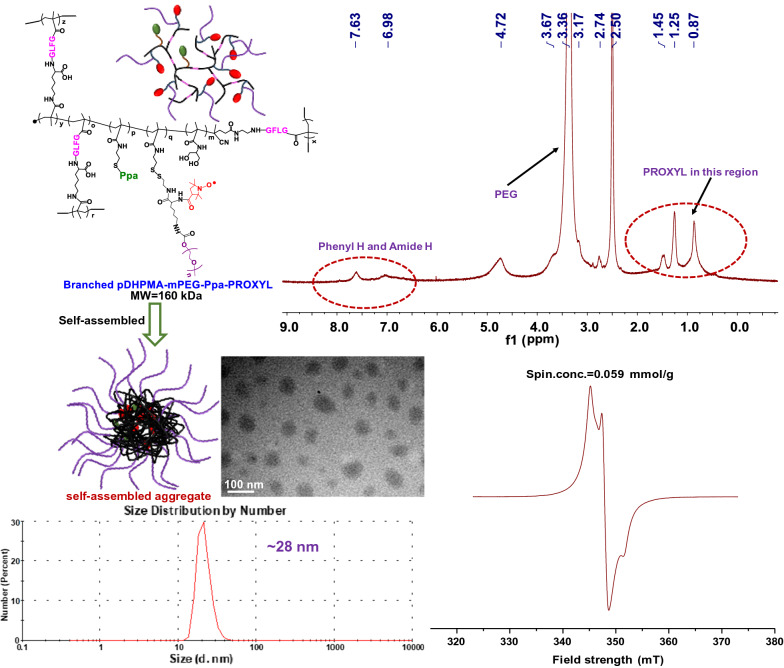
Basic physical and chemical properties of Branched pDHPMA-mPEG-Ppa-PROXYL including chemical structure, ^1^HNMR, skematic self-assembly structure, size and morphology (~ 28 nm, by TEM and DLS) and Spin concentration (0.059 mmol/g, by EPR)

In this study, we designed and synthesized a novel nitroxide radicals-based mCA, Branched pDHPMA-mPEG-Ppa-PROXYL, which was derived from an enzyme/GSH sensitive PEGylated branched DHPMA copolymer as the macromolecular skeleton. Amphiphilic Branched pDHPMA-mPEG-Ppa-PROXYL could self-assembly into nano-sized aggregates in an aqueous environment. As shown in Fig. 1, the hydrophilic components formed the outer layer to encapsulate hydrophobic nitroxides (PROXYL) inside, thereby enhancing the stability of PROXYL. Enhanced stability combined with the macromolecular effects, including long blood circulation, increased nitroxides concentration and slow molecular rotation, rendered this nitroxides-based metal-free mCA to be used for MR imaging of multiple targets in vivo including tumor, liver, kidney and cardiovascular system (heart and aortaventralis). In particular, to our knowledge, this is the first report on nitroxides-based MRI CAs for imaging the cardiovascular system.

## Materials and methods

Materials, methods, synthesis of the PROXYL-based branched biodegradable mORCA (Branched pDHPMA-mPEG-Ppa-PROXYL, Additional file 1: Scheme S1) and its characterizations (Additional file 1: Table S1) were supplemented in the Supporting Information. In vitro or vivo toxicity, blood compatibility test and cell uptake experiment methods were also placed in the Supporting Information.

### Animal and tumor models

All animals were fed in a control room. 7 × 10^5^ 4T1 cells were inoculated subcutaneously in the dorsal side of each mouse. When the tumor reached 100 mm^3^, all experimental mice were randomly divided for MR imaging and other experimental studies.

### In vitro relaxivity and in vivo MR imaging

The longitudinal relaxivity of Branched pDHPMA-mPEG-Ppa-PROXYL in phosphate buffer saline (PBS) was measured using a clinical Siemens 3.0 T MRI scanner. Branched pDHPMA-mPEG-Ppa-PROXYL at different concentrations of PROXYL (0, 0.17, 0.34, 0.68, 1.35, 2.7, 5.4, 10.8 mM) was dissolved in 0.1 M PBS, and MR signals of the prepared solutions were scanned by T1 SE sequence with scanning parameters: TE = 6.9 ms, TR = 20, 30, 50, 70, 90, 125, 150, 175, 200, 300, 400, 500, 700, 850, and 1000 ms, Fov = 200 mm, slice thickness = 1.0 mm, and matrix dimensions = 256 × 256. The corresponding 1/T1 values were obtained from their T1-weighted MR images. The value of relaxivity (*r*_*1*_) was calculated by plotting 1/T1 as a function of different PROXYL concentrations. In addition, 3-Carboxy-PROXYL (3-CP) at the same concentration in 0.1 M PBS was used as a control.

Twenty 8–10 weeks healthy female BALB/c mice were randomly divided into four groups (5 in each group, 20 ± 2 g). Another ten female BALB/c mice of tumor-bearing (20 ± 2 g, 8–10 weeks) were also randomly divided into two groups (n = 5). The MR signals of main organs (heart, liver and kidney), aortaventralis and tumor in the body at different time points were obtained via a clinical Siemens 3.0 T MRI scanner. A mouse coil was employed (Shanghai Chenguang Medical Technology Co., Ltd., model: CG MUC23 H300 AS with an eight-channel phased array structure), and the T_1_ mapping sequence was used for coronal scanning. The scan sequence was: TR = 15 ms, TE = 2.04 ms, flip angle = 5°/26 °, slices = 10, slice space = 0.4 mm, thickness = 2.0 mm, and Fov = 156 × 156 mm. Scanning was executed at pre-injection, 5, 10, 15, 20, 25, and 30 min after the injection of Branched pDHPMA-mPEG-Ppa-PROXYL and 3-CP at a dose of 0.135 mmol/kg PROXYL to obtain images of each organ, aortaventralis and tumor. The MATLAB software [45, 46] was used to analyze the T_1_ values of each organ, aortaventralis and tumor before and after enhancement.

### In vivo metabolism

Ten female healthy BALB/c mice (8–10 weeks, 20 ± 2 g) were randomly divided into two groups (n = 5), and Branched pDHPMA-mPEG-Ppa-PROXYL and 3-CP were injected through the tail vein at a dose of 0.135 mmol/kg PROXYL, respectively. 50 µL of blood samples were collected through the fundus vein at time points of 3 min, 6 min, 9 min, 12 min, 15 min, 30 min, 1 h, 2 h, 4 h, 8 h, and 24 h after injection. The blood samples were centrifuged (10,000*g* × 5 min) and the serum supernatants were collected. The spin concentration (the nitroxides content) of each serum sample at different time points was measured by electronic paramagnetic resonance (EPR).

## Results and discussion

### Preparation and characterization of Branched pDHPMA-mPEG-Ppa-PROXYL

According to our previous studies, Gd(III)-based mCAs constructed from DHPMA copolymers with a branched structure could make full use of the macromolecular effect to achieve high relaxivities and great in vivo MR imaging. Therefore, in this study, we prepared a branched DHPMA copolymer whose each branched chain contains an enzyme sensitive GFLG peptide for the construction of a novel biodegradable branched PROXYL-based mORCA (Branched pDHPMA-mPEG-Ppa-PROXYL, as shown in Additional file 1: Scheme S1) in the hope of effectively improving the relaxivity and in vivo MR imaging of PROXYL via significant macromolecular effect. In addition, its detailed characterization is listed in Additional file 1: Table S1.

Firstly, we prepared a PEGylated PROXYL derivative functionalized by dithiopyridyl (PTE) (PTE-mPEG-PROXYL, as shown in Additional file 1: Scheme S1) in a similar method we reported [35], and PTE-mPEG-PROXYL has the following functions: (1) the PTE groups can react with thiols (thiol-disulfide exchange reaction) to covalently introduce PROXYL derivatives onto the macromolecular material, forming the corresponding mORCA; (2) Due to strong hydrophobicity of PROXYL, its introduction will weaken the water solubility of the mORCA, so we adopted PEGylation to balance hydrophobicity and hydrophilicity in the structure. PTE-mPEG-PROXYL contains the same amount of mPEG_2000_ and PROXYL, therefore, after covalently connecting with the macromolecule, it can achieve a good water-solubility and obtain a higher content of PROXYL, so as to ensure full realization of the multiplier effect; (3) Additionally, PEGylation can further improve MW of the mORCA and enhance its biocompatibility and in vivo stability.

On the other hand, in our previous study, a branched DHPMA copolymer with a short enzyme-sensitive GFLG peptide in each branch chain (Branched pDHPMA-SH, as shown in Additional file 1: Scheme S1) was prepared *via* RAFT polymerization of DHPMA, PTEMA, MA-GFLGK-MA and MA-GFLG-NH-CTA induced by VA044. The Gd-based mCA derived from this copolymer displayed excellent relaxation performance both in vivo and in vitro, and also good biodegradability and low side effects [44]. Inspired by this result, we selected Branched pDHPMA-SH as a macromolecular carrier to construct a novel PROXYL-based mORCA (Branched pDHPMA-mPEG-Ppa-PROXYL, as shown in Additional file 1: Scheme S1). The synthesis process had two steps: (1) First, maleimide-functionalized pyropheophorbide-α (Ppa-Maleimide) as a fluorescent probe was covalently introduced *via* thiol-ene click chemistry, and the input quantity of Ppa-Maleimide did not exceed 1 % of the total amount of raw material, which can meet the requirements of fluorescence imaging but does not affect the water solubility of the final copolymer; (2) Next, the PEGylated PROXYL derivative was covalently introduced *via* disulfide-thiol exchange reaction. The structure of the final copolymer was confirmed by ^1^HNMR (Additional file 1: Fig. S1), and gel permeation chromatography (GPC) measured its MW as 160 kDa (Additional file 1: Table S1). EPR analysis showed that the copolymer had paramagnetism and the spin concentration (nitroxides content) was 0.059 mmol/g (Additional file 1: Fig. S2). According to our design ideas, Branched pDHPMA-mPEG-Ppa-PROXYL has amphipathy and can self-assemble into an aggregate with a certain nanometer-size in water. We analyzed the particle size and morphology of the copolymer by dynamic light scattering (DLS) and transmission electron microscopy (TEM), and the results showed that Branched pDHPMA-mPEG-Ppa-PROXYL could form a self-assembled aggregate at a particle size of 28 nm (Additional file 1: Fig. S3–4). The amino acid analysis result (Additional file 1: Table S2) showed that the molar ratio of Gly/Phe/Leu was ca. 1.4/1.7/1, which indicated that the short peptide GFLG was introduced into the mORCA. Additionally, from DLS (Additional file 1: Fig. S5), the zeta potential of the final copolymer was ca. 0 mV, which indicated that the surface of Branched pDHPMA-mPEG-Ppa-PROXYL was electrically neutral, so it can prevent adsorption of proteins in the blood, resulting in a long retention time in the blood. All above chemical properties will result in good in vitro and in vivo performance of the mCA. First, its big molecular size and high content of nitroxides will help to improve the relaxivity via macromolecular effects including slow molecular rotation and accumulative effect. Second, it appears to be a stable amphiphilic nano-structure in aqueous environment, which is beneficial to enhance the in vivo stability of PROXYL by effective protection. The above two points, together with the inherent characteristics of macromolecules, make Branched pDHPMA-mPEG-Ppa-PROXYL very promising to produce excellent MR imaging effect in vivo.

### Relaxivity of Branched pDHPMA-mPEG-Ppa-PROXYL

**Fig. 2:**
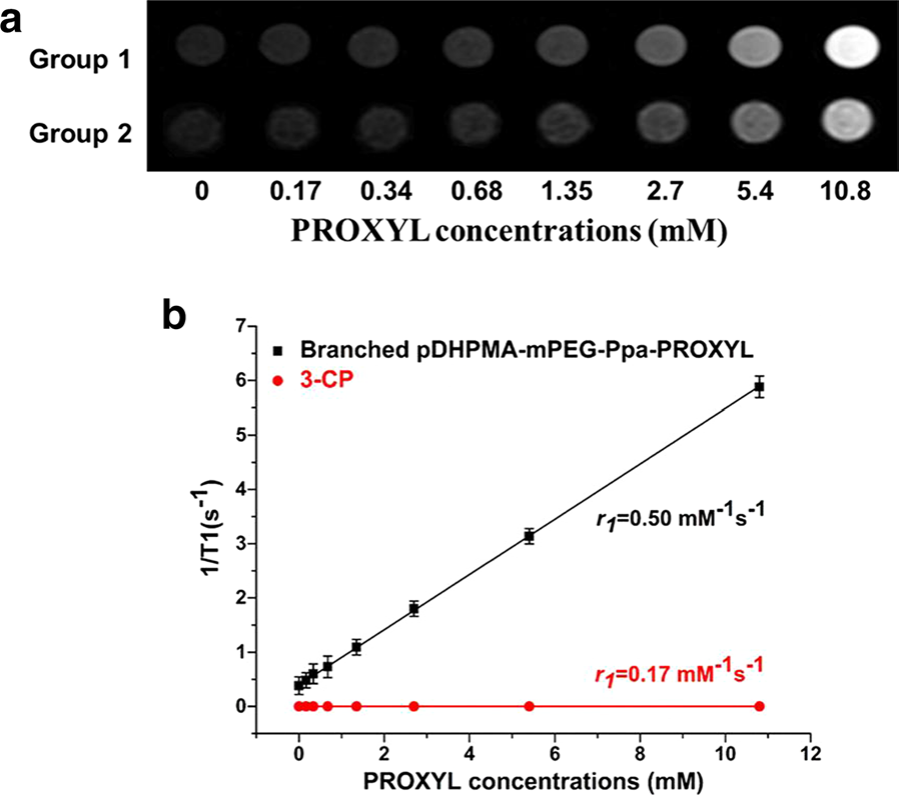
**a** T1-weighted MRI images of copolymers with gradient PROXYL concentrations. **b** The relaxivity curve of Branched pDHPMA-mPEG-Ppa-PROXYL (Group 1) and 3-CP (Group 2) in the 3.0 T scanner

A clinical Siemens 3.0 T MRI scanner was used to measure the longitudinal relaxivity (*r*_*1*_) of Branched pDHPMA-mPEG-Ppa-PROXYL with 3-CP as a control. Bright signals were seen from the Branched pDHPMA-mPEG-Ppa-PROXYL samples in MRI images, and these signals were more intense than those from 3-CP at the equivalent PROXYL concentration (Fig. 2a). As shown in Fig. 2b, the *r*_*1*_ values were calculated by plotting 1/T1 with the gradient concentration of Branched pDHPMA-mPEG-Ppa-PROXYL and 3-CP. The in vitro relaxation efficiency (*r*_*1*_ = 0.50 mM^− 1^ s^− 1^) of Branched pDHPMA-mPEG-Ppa-PROXYL was significantly higher than that (*r*_*1*_ = 0.17 mM^− 1^ s^− 1^) of 3-CP.

### In vivo major organ and aortaventralis imaging

**Fig. 3:**
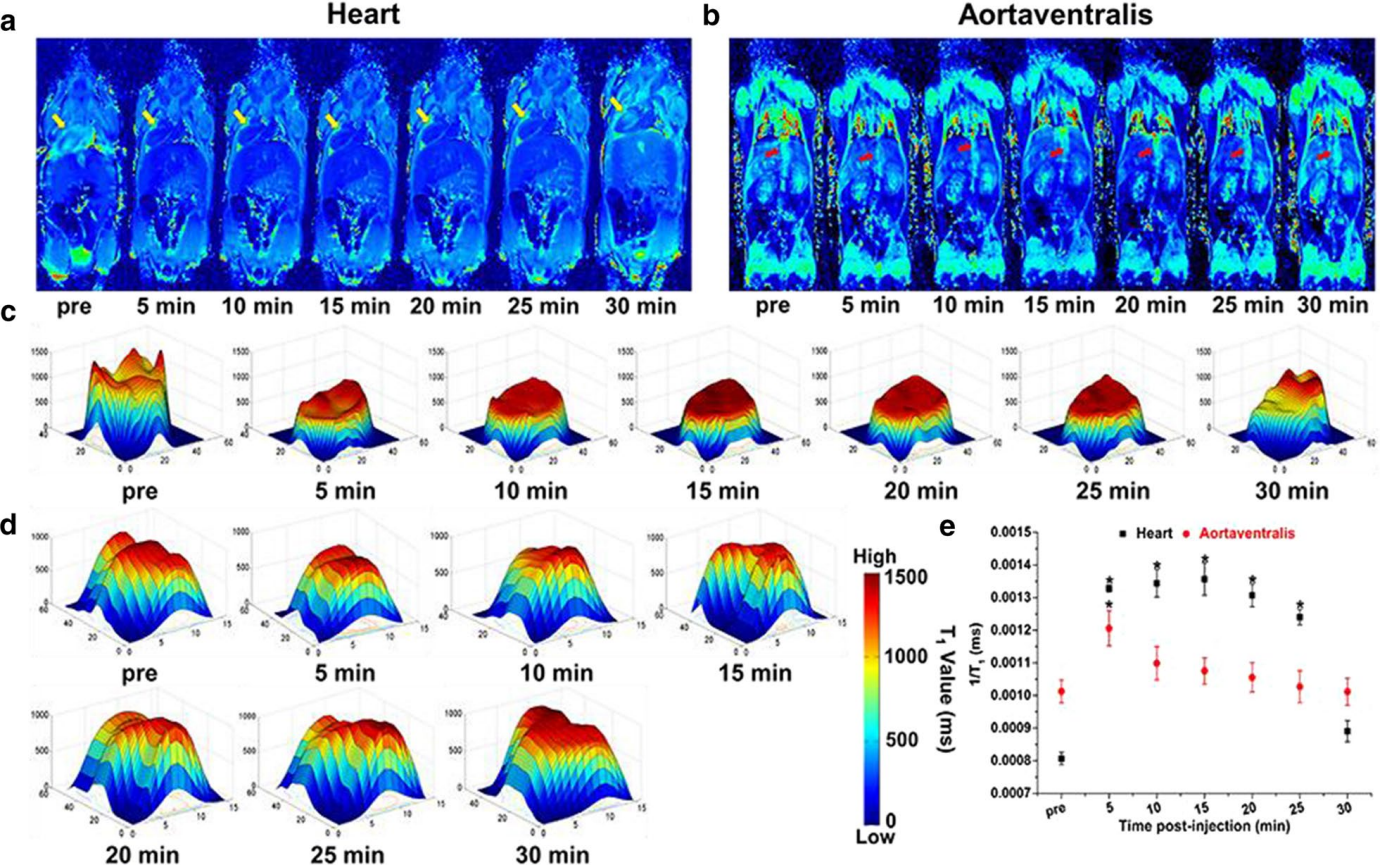
Representative T_1_ mapping imaging of heart **a** and aortaventralis **b** of one mouse after injection of Branched pDHPMA-mPEG-Ppa-PROXYL. The heart and aortaventralis were labeled with yellow and red arrow, respectively. The darker blue signals in the heart and aortaventralis suggest sharper enhancement in the MR images. The corresponding T_1_ values were spatially displayed in the heart **c** and aortaventralis **d** after injection of Branched pDHPMA-mPEG-Ppa-PROXYL at different durations. **e** The 1/T_1_ values for the heart and aortaventralis were quantitatively analyzed (n = 5, *p < 0.05, compared with pre-injection)

A clinical 3T MRI scanner was used to detect MRI T_1_ mapping signals of Branched pDHPMA-mPEG-Ppa-PROXYL in living mice to evaluate its suitability as an MRI CA. A small molecule, 3-CP, was employed as a control. As shown in Fig. 3a, the T_1_ mapping signals in the heart were intensified significantly at 5 min after injection, and the intensified trend continued to reach a peak at 15 min. After that, the signals became slowly weakened, while dark blue signals were still detectable at 30 min after injection of the contrast agent. Additionally, T_1_ mapping signals in the aortaventralis appeared to be strengthened during the initial 5 min after injection and the signal peak was reached at 5 min. The degree of T_1_ mapping signal enhancement started to decrease after 5 min, and at 30 min the signal was observed to reduce to the same level as that before injection (Fig. 3b). Furthermore, we performed quantitative analysis of T_1_ mapping images in the heart and aortaventralis *via* the T_1_ value. In the heart (Fig. 3c), the T_1_ value increased in the initial few minutes after injection of the mCA and reached the enhancement peak at 15 min. An increase by about 168 % in the T_1_ value was achieved in comparison with the level before enhancement (Fig. 3e). After 15 min, the T_1_ value gradually decreased. The increasing pattern was also seen for the T_1_ value in the aortaventralis (Fig. 3d), while the change gradient was much steeper and the value peaked at 5 min with an increase by about 120 % compared to the level before enhancement (Fig. 3e).

**Fig. 4:**
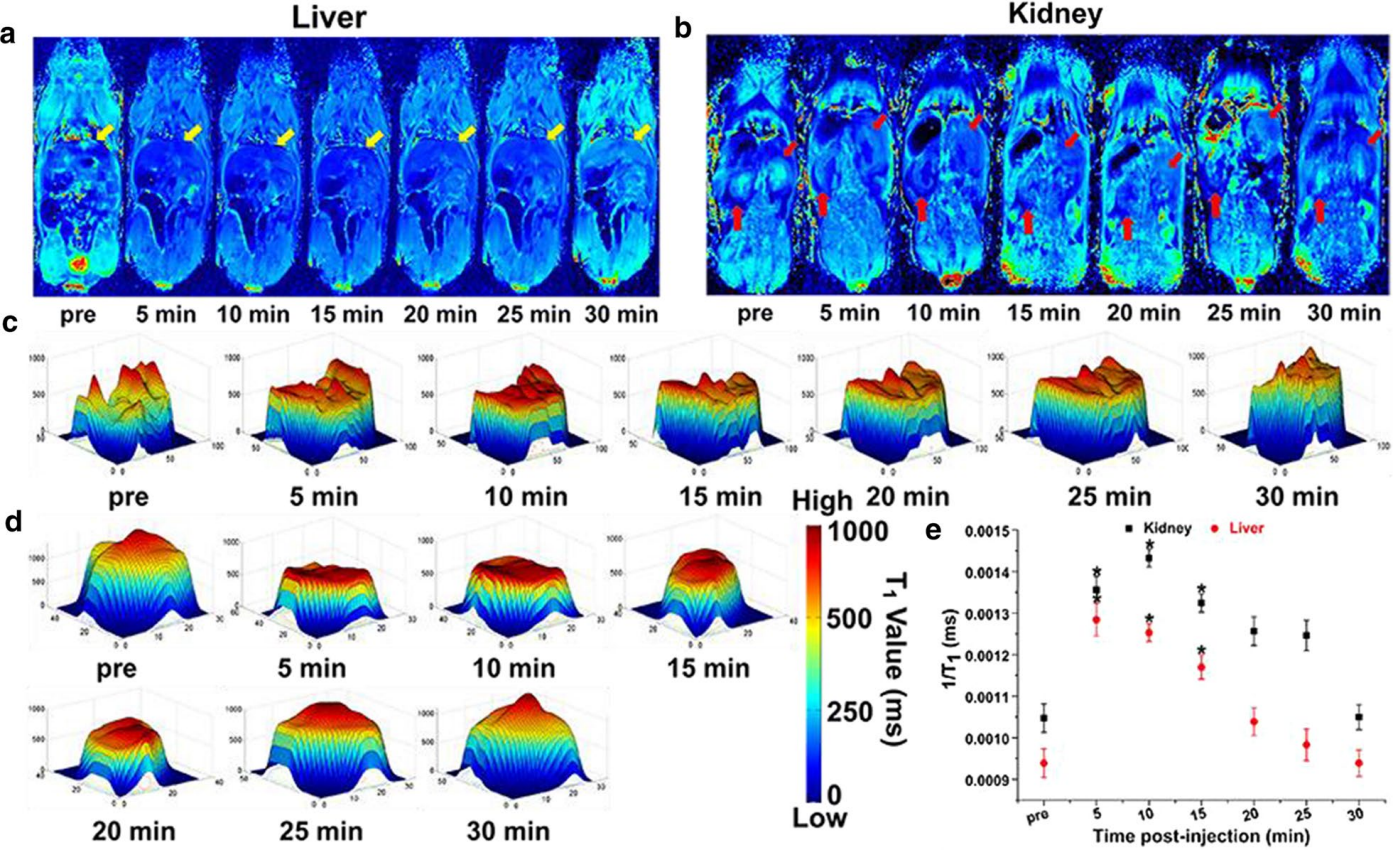
Representative T_1_ mapping imaging of liver **a** and kidney **b** of one mouse after injection of Branched pDHPMA-mPEG-Ppa-PROXYL. The liver and kidney were labeled with yellow and red arrow, respectively. The darker blue signals in the liver and kidney suggest sharper enhancement in the MR images. The corresponding T_1_ values were spatially displayed in the liver **c** and kidney **d** after injection of Branched pDHPMA-mPEG-Ppa-PROXYL at different durations. **e** The 1/T_1_ values for the liver and kidney were quantitatively analyzed (n = 5, *p < 0.05, compared with pre-injection)

In the liver (Fig. 4a), the T_1_ mapping signals began to increase and reached the enhancement peak at 5 min after injection. After 5 min, the signals began to decline continuously, and recovered at the pre-injection level in 30 min. The T_1_ mapping signals appeared to increase in the kidney (Fig. 4b) after 5 min and it reached an enhancement peak at 10 min. The enhancement was noticed to last until 25 min after injection of the mCA, and the signals returned to the pre-injection level at about 30 min. Quantitative analysis of T_1_ mapping images in the liver and kidney *via* the T_1_ value. We found that the T_1_ value in the liver (Fig. 4c) increased significantly within 5 min post-injection of the mCA and peaked at 5 min with the same trend as the T_1_ mapping signals. The T_1_ value was enhanced by about 137 % compared to the level before enhancement (Fig. 4e). The T_1_ value then gradually decreased and reduced to the pre-injection level at 30 min. In the kidney (Fig. 4d), the T_1_ value increased to a peak at 10 min with an enhancement of about 136 % compared to the level before enhancement (Fig. 4e). Both T_1_ mapping signals and the T_1_ value in the organs (heart, liver and kidney) and aortaventralis displayed negligible changes after injection of 3-CP (Additional file 1: Fig. S6–S8). This can be explained as: (1) Branched pDHPMA-mPEG-Ppa-PROXYL has a higher relaxation efficiency in vitro; (2) Branched pDHPMA-mPEG-Ppa-PROXYL can form self-assembled aggregates with a larger particle size in the aqueous environment, and the hydrophobic nitroxides are encapsulated inside a hydrophilic outer layer of the self-assembled aggregates, so it can provide relatively long and stable MRI enhancement in the heart, liver, kidney and aortaventralis. At the same time, the T_1_ mapping signals and T_1_ value of the kidney increased, suggesting that Branched pDHPMA-mPEG-Ppa-PROXYL is mainly metabolized by the kidney, thereby ensuring its safety.

### In vivo tumor imaging

Based on the above results from in vitro and in vivo MR imaging, we further studied the imaging efficacy of Branched pDHPMA-mPEG-Ppa-PROXYL at the tumor site. We also used T_1_ mapping sequences to scan and observe the MRI enhancement in tumor-bearing mice at various time points. The imaging efficacy was quantified by T_1_ value, and 3-CP was also set up as a control group for the same scan.

**Fig. 5:**
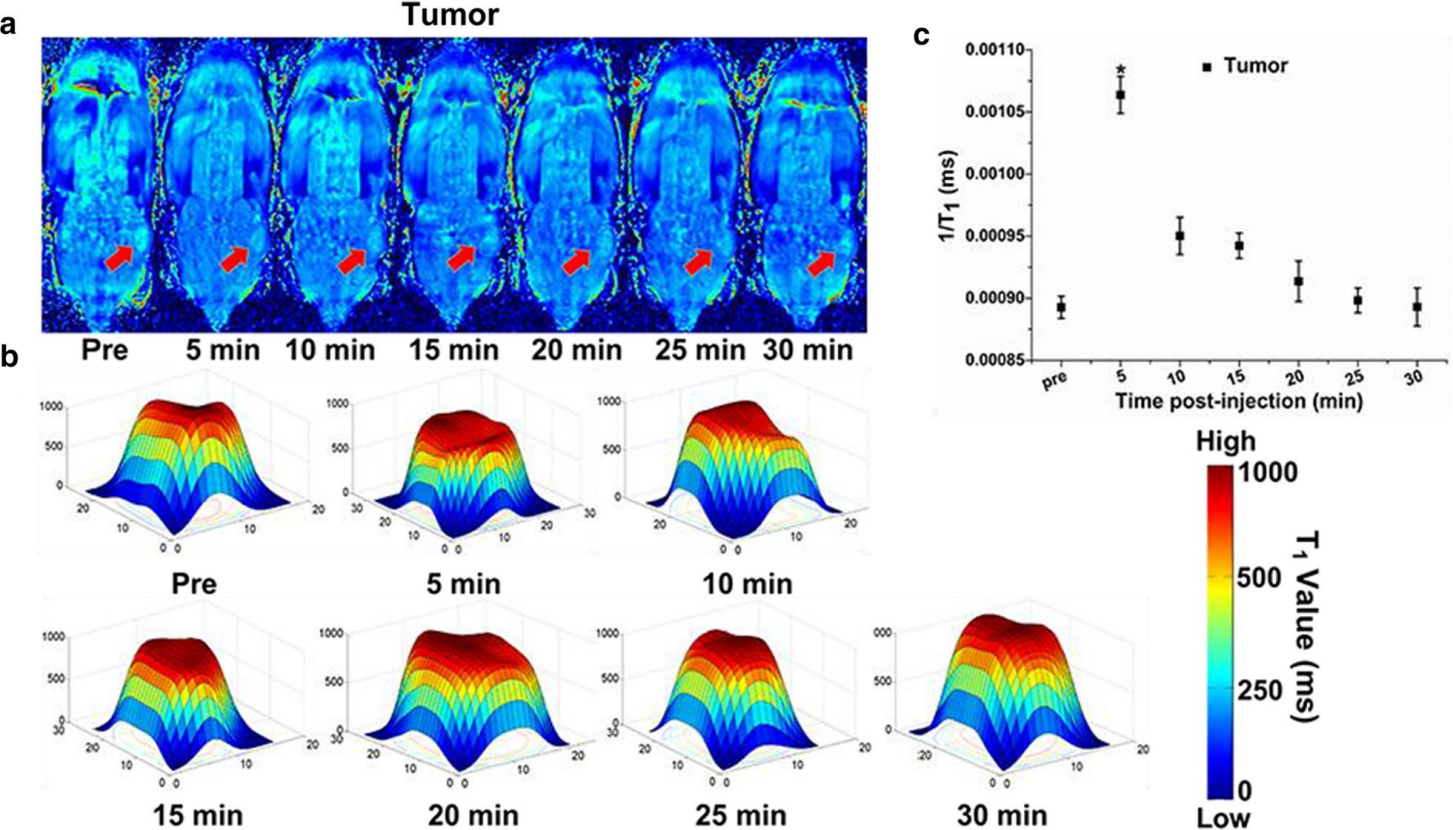
Representative T_1_ mapping imaging of tumor of one mouse by Branched pDHPMA-mPEG-Ppa-PROXYL **a**. The tumor was labeled with red arrow, and darker blue signals in the tumor suggest sharper enhancement in the MR images. The corresponding T_1_ values were spatially displayed in the tumor after injection of Branched pDHPMA-mPEG-Ppa-PROXYL **b** at different durations. **c** The 1/T_1_ values of two tumor groups were quantitatively analyzed (n = 5, *p < 0.05, compared with pre-injection)

After injection of the contrast agents, the T_1_ mapping signal at the tumor site began to strengthen within 5 min and reached the enhancement peak (Fig. 5a), and then began to decrease continuously, returning to the pre-injection level at 30 min. Quantitative analysis of the above tumor T_1_ mapping images by T_1_ value (Fig. 5b) shows that Branched pDHPMA-mPEG-Ppa-PROXYL reached the enhancement peak of the tumor site at ca. 5 min. The T_1_ value increased by about 144 % (Fig. 5c), showing a very high enhancement effect, this result was also consistent with the in vitro relaxivity results. After 5 min, the T_1_ value gradually decreased and returned to the pre-injection level at ca. 30 min. Subsequently, the small molecular 3-CP group was scanned by the same method as described above, and no significant increase in the T_1_ mapping signal at the tumor site was found (Fig. S9). It is known that the tumor tissue contains a higher concentration of reducing substances (such as GSH) than normal tissues, and this will seriously affect the MR imaging efficacy of Branched pDHPMA-mPEG-Ppa-PROXYL in tumors. However, the passive targeting ability to tumors and the protection of PROXYL by the amphiphilic structure allowed Branched pDHPMA-mPEG-Ppa-PROXYL to provide a great MRI enhancement in tumor tissues.

### In vivo metabolism of PROXYL

To measure the residence time of nitroxides released from Branched pDHPMA-mPEG-Ppa-PROXYL in the blood, we injected Branched pDHPMA-mPEG-Ppa-PROXYL and 3-CP as a control into normal mice via tail vein at a dose of 0.135 mmol PROXYL/kg, and the blood samples at the same volume were collected at different pre-established time points to analyze the nitroxides concentrations in these blood samples by EPR.

**Fig. 6:**
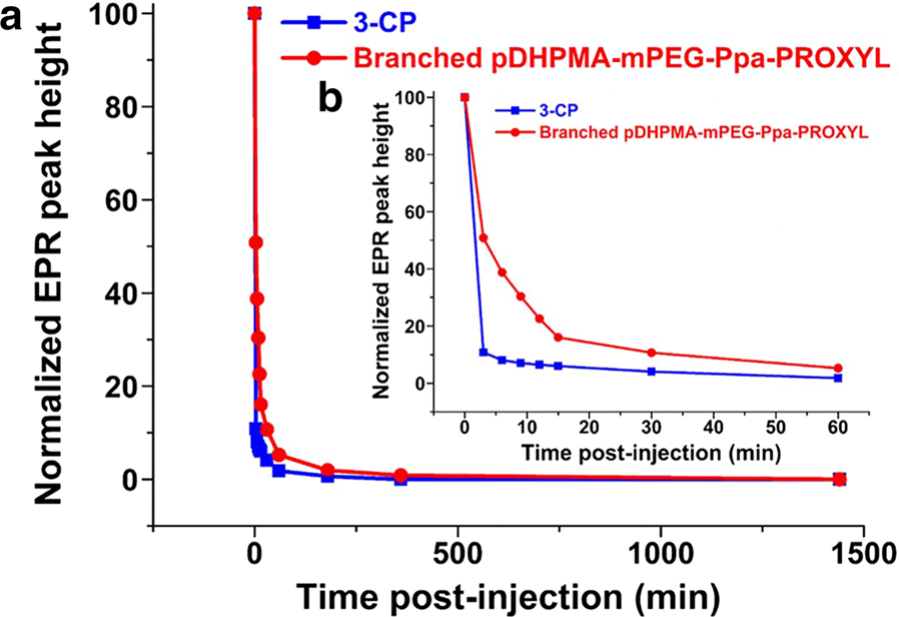
**a** Nitroxides concentration distribution in vivo for 24 h after injection of Branched pDHPMA-mPEG-Ppa-PROXYL and 3-CP and **b** nitroxides concentration changes within 1 h

The temporal changes of nitroxides concentrations in the blood of mice were shown in Fig. 6. It was observed that the PROXYL concentrations of 3-CP in the blood rapidly decreased and they were below the detection limit at 1 h after the injection, indicating that the small molecule, 3-CP, was rapidly eliminated by reducing substances in the blood. However, the PROXYL concentration reduced much slowly in the Branched pDHPMA-mPEG-Ppa-PROXYL group. In the initial post-injection period, the nitroxides concentration was high and had more contact with reducing substances in vivo, therefore, the PROXYL concentration in the Branched pDHPMA-mPEG-Ppa-PROXYL group displayed a similar decreasing trend as that in the 3-CP group. However, as the concentration of nitroxides concentration gradually decreased, the macromolecular effects gradually started to become important, so the decreasing trend of nitroxides concentration became slower. As a result, the reduction rate of the nitroxides concentration in the Branched pDHPMA-mPEG-Ppa-PROXYL group was much less steeper than that in the 3-CP group, and the nitroxides were still detectable until 6 h after injection.

The macromolecular structure of Branched pDHPMA-mPEG-Ppa-PROXYL plays a critical role in the extended circulation time of nitroxides: (1) The nitroxides are protected in the hydrophobic core of self-assembled aggregates, which results in a decrease in the interaction rate between endogenous reducing substances in the blood and nitroxides, thereby, nitroxides residues are still detectable up to 6 h post-injection; (2) As a macromolecule, Branched pDHPMA-mPEG-Ppa-PROXYL itself has a long blood retention time. A long blood retention time of Branched pDHPMA-mPEG-Ppa-PROXYL allows great accumulation of this mCA in the major organs, aortaventralis and tumor tissues, thus, enhancements of imaging contrast in these tissues/organs are achieved.

### Cellular uptake

**Fig. 7:**
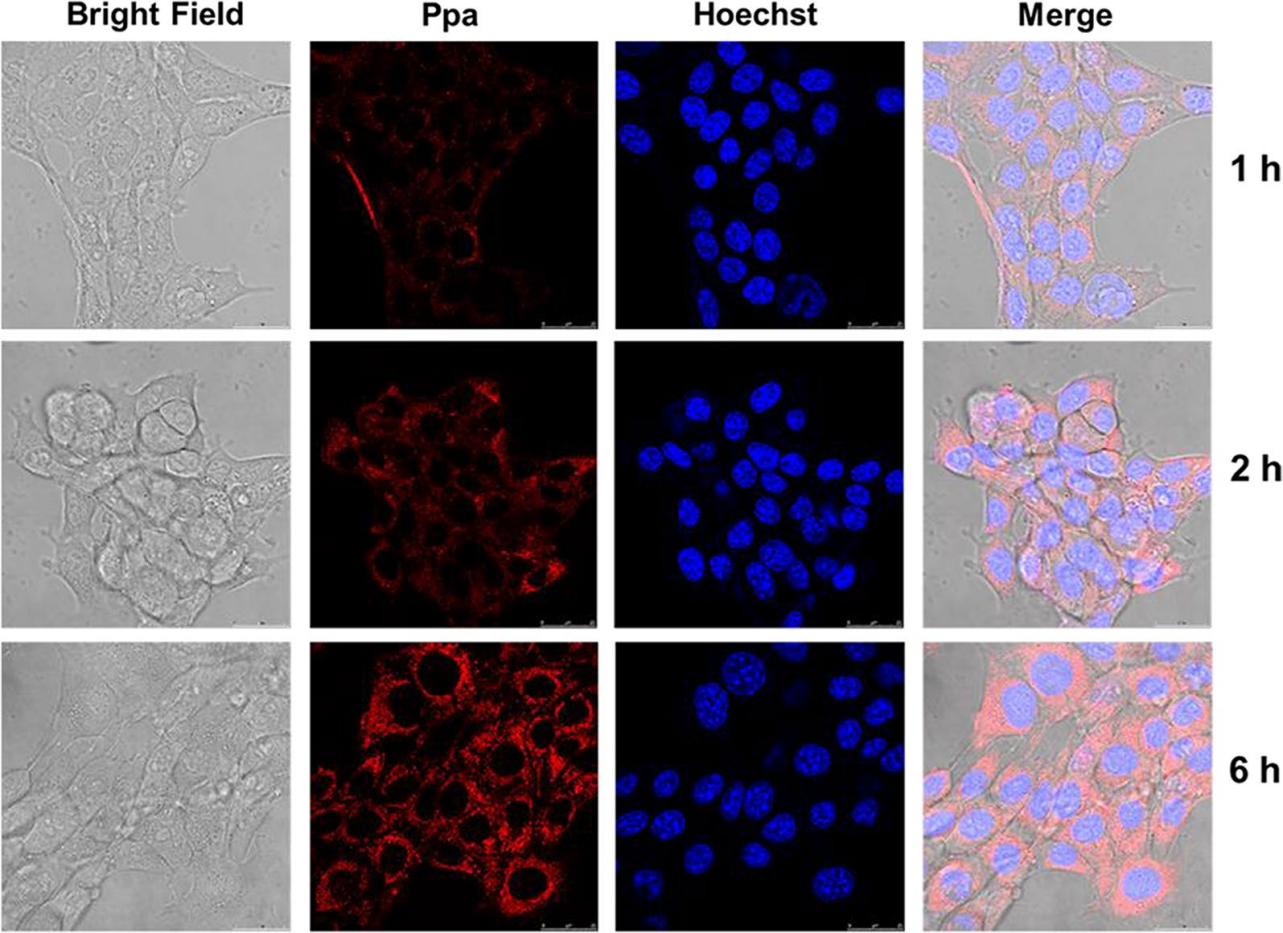
CLSM images for uptake of Branched pDHPMA-mPEG-Ppa-PROXYL by 4T1 cells at 1 h, 2 h, and 6 h Branched pDHPMA-mPEG-Ppa-PROXYL (red fluorescence) distributed in the cytoplasm without entering the nucleus (blue fluorescence), and the cells have a time-dependent behavior for the cellular uptake of this mORCA. Scale bar: 25 μm

CLSM was used to study the uptake behavior and distribution of Branched pDHPMA-mPEG-Ppa-PROXYL in 4T1 cells. It was observed that Branched pDHPMA-mPEG-Ppa-PROXYL started to enter 4T1 cells at 1 h after incubation, and the cytoplasmic fluorescence intensity at 6 h after incubation was significantly stronger than at at 1 and 2 h after incubation. Branched pDHPMA-mPEG-Ppa-PROXYL accumulated in the cytoplasm (red fluorescence) and did not enter the nucleus (blue fluorescence) (Fig. 7). The experimental results showed that Branched pDHPMA-mPEG-Ppa-PROXYL could be taken up by 4T1 cells and distributed in the cytoplasm without entering the nucleus, and the cells have a time-dependent behavior for the cellular uptake of this mORCA.

### In vitro cytotoxicity

**Fig. 8:**
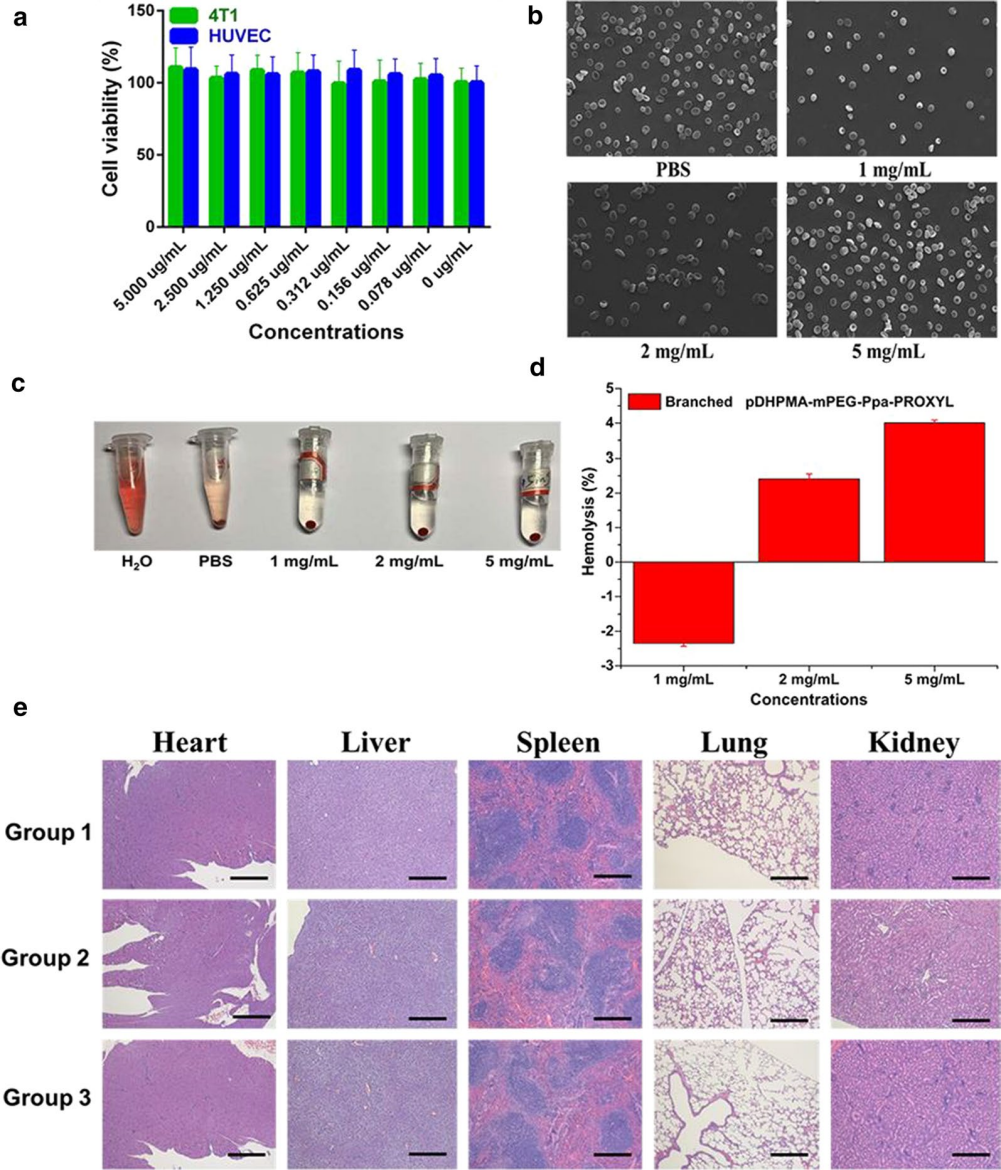
**a** Cell viability of 4T1 cells and HUVEC cells after incubation with Branched pDHPMA-mPEG-Ppa-PROXYL for 24 h. **b** The effects of PBS, 1 mg/mL, 2 mg/mL, and 5 mg/mL Branched pDHPMA-mPEG-Ppa-PROXYL on erythrocyte aggregation and morphology. **c** Red blood cell hemolysis of Branched pDHPMA-mPEG-Ppa-PROXYL. **d** Hemolysis rates of Branched pDHPMA-mPEG-Ppa-PROXYL at different concentrations. **e** Histological images of mice on day 1 after injection of saline (Group 1), 3-CP (Group 2) and Branched pDHPMA-mPEG-Ppa-PROXYL (Group 3). Scale bar: 10 μm

As shown in Fig. 8a, after incubation with Branched pDHPMA- mPEG-Ppa-PROXYL (from 0 to 1 mg/mL) for 24 h, the viability of 4T1 and HUVEC cells showed no significant difference, and the concentration of PROXYL had negligible impact on the cell viability, which indicated that Branched pDHPMA-mPEG-Ppa PROXYL had no obvious toxicity to 4T1 and HUVEC cells. This could be due to a neutral surface charge of Branched pDHPMA-mPEG-Ppa PROXYL and a biodegradable macromolecular structure since the short peptide linker GFLG in the structure could be cleaved by cathepsin B in the cytoplasm.

### Blood compatibility evaluation

We evaluated hemolysis induced by Branched pDHPMA-mPEG-Ppa-PROXYL by observing the red blood cell (RBC) morphology via a SEM. As shown in Fig. 8b, after Branched pDHPMA-mPEG-Ppa-PROXYL at different concentrations was incubated with RBCs, the morphology of RBCs did not change significantly, and no red blood cells were aggregated. At a high magnification (1000x), the membrane surface of RBCs was smooth and intact with a normal biconcave disk-like structure. Compared with the PBS control group, there were no obvious changes with any abnormal structures such as collapse and rupture. Therefore, Branched pDHPMA-mPEG-Ppa-PROXYL did not cause any changes in the morphology and aggregation of RBCs and they had great biological safety.

After adding Branched pDHPMA-mPEG-Ppa-PROXYL to the whole blood, we measured the hemolysis with pure water and PBS as controls. The results showed that Branched pDHPMA-mPEG-Ppa-PROXYL did not cause hemolytic reaction at each concentration (Fig. 8c), which indicated that Branched pDHPMA-mPEG-Ppa-PROXYL had great blood compatibility and excellent biological safety. The degree of hemolysis is often quantified with the amount of hemoglobin released from RBCs after they are ruptured. The microplate reader was used to measure the absorbance of the sample at 540 nm because the absorption wavelength of hemoglobin is 540 nm and the hemoglobin content in the samples can be determined from the absorbance. The ASTM standards suggest that the hemolysis level for blood compatibility should be less than 5 %. After we incubated Branched pDHPMA-mPEG-Ppa-PROXYL at 1.0, 2.0, and 5.0 mg/mL for 12 h, the hemolysis level at each concentration of the mCA was less than 5 % (Fig. 8d), and no significant difference was observed compared with PBS. Therefore, Branched pDHPMA-mPEG-Ppa-PROXYL had excellent blood compatibility.

### In vivo toxicity evaluation

In order to evaluate the biological safety of Branched pDHPMA-mPEG-Ppa-PROXYL as a safe MR contrast agent, we further observed the toxic effect of the contrast agent in vivo. After 24 h of injection, the mice did not display obvious acute toxic and side effects (such as bleeding, motor ataxia, death, etc.). All these mice were killed and main organs (heart, liver, spleen, lung and kidney) were dissected for pathological analysis and blood samples were collected for routine blood and blood biochemical index analysis, such as RBCs, white blood cells (WBCs), hemoglobin (HGB), hematocrit (HCT), platelet (PLT), mean platelet volume (MPV), aspartate aminotransferase (AST), alanine aminotransferase (ALT), creatinine (CREA), alkaline phosphatase (ALP), glutamyltransferase (GGT) and blood urea nitrogen (BUN). The analysis results showed that compared with the 3-CP and saline control groups, no obvious tissue damage and abnormalities in histopathology (Fig. 8e) and blood chemistry indexes (Additional file 1: Figs. S11, 12) (such as inflammation, necrosis, atrophy, atrophy, tissue deformation, etc.) were seen in the mCA group. The experimental results indicated that at the MR imaging dose, Branched pDHPMA-mPEG-Ppa-PROXYL was non-toxic to the blood, organs and tissues of living animals.

## Conclusions

Herein, we designed and prepared a water-soluble biodegradable branched polymeric nitroxides as a novel metal-free mCA (Branched pDHPMA-mPEG-Ppa-PROXYL) via covalently conjugation of a PEGylated PROXYL derivative onto an enzyme sensitive degradable branched pDHPMA. Its molecular weight (MW) and nitroxide radical content are 160 kDa and 0.059 mmol/g, respectively. Additionally, it is amphiphilic and can form a nanoscale (~ 28 nm) self-assembled aggregate in a physiological environment, which is beneficial to prolong the circulation time in vivo; enhance the targeting of specific tissues (e.g. tumor); and reduce the contact between PROXYL in hydrophobic core and reducing substances in vivo thus enhancing its stability. Thanks to the macromolecular effects and a higher nitroxides content, in vivo and in vitro studies showed that Branched pDHPMA-mPEG-Ppa-PROXYL was superior to 3-CP in many aspects including the in vitro longitudinal relaxivity, in vivo stability of PROXYL and the MR imaging efficacy. At the same time, it did not show obvious toxicity in vivo. It should be pointed out that compared with all CAs of the same kind reported previously, Branched pDHPMA-mPEG-Ppa-PROXYL could not only work in tumors and major organs (e.g. liver and kidney), but also provide MR imaging enhancements in the cardiovascular system. Therefore, Branched pDHPMA-mPEG-Ppa-PROXYL is a safe and efficient metal-free mCA for multi-object MRI in vivo. However, the in vitro relaxivity of Branched pDHPMA-mPEG-Ppa-PROXYL in this study is not high enough. The content of nitroxides in the macromolecular structure could be further increased without compromising its stable nanostructure in water environment, molecular weight and water solubility, thereby increasing the relaxivity of this mCA, in order to achieve better contrast imaging of in vivo tissues.

## Supplementary Information

**Additional file 1:** **Scheme S1.** Preparation of Branched pDHPMA-mPEG-Ppa-PROXYL. **Table S1.** Properties and characterizations of copolymers. **Table S2.** Contents of amino acids in the copolymers (wt%). **Fig. S1.** ^1^H NMR spectra of **a** Branched pDHPMA-SH and **b** Branched pDHPMA-mPEG-Ppa-PROXYL (400 MHz, *d*_6_-DMSO as solvent). **Fig. S2.** The EPR spectrum of Branched pDHPMA-mPEG-Ppa-PROXYL. **Fig. S3.** The particle size of Branched pDHPMA-mPEG-Ppa-PROXY (ca. 28 nm, DLS). **Fig. S4.** TEM images of Branched pDHPMA-mPEG-Ppa-PROXYL. **Fig. S5.** Zeta Potential of Branched pDHPMA-mPEG-Ppa-PROXYL (ca. 0 mV, DLS). **Fig. S6.** T_1_ mapping of the heart after injection of 3-CP. **Fig. S7.** T_1_ mapping ofthe aortaventralis after injection of 3-CP. **Fig. S8.** T_1_ mapping of the liver and kidney after injection 3-CP. **Fig. S9.** T_1_ mapping of the tumorsite after 3-CP injection. **Fig. S10.** MRI signals of Branched pDHPMA-mPEG-Ppa-PROXYL **a** and DTPA-Gd **c**. **Fig. S11.** Routine blood tests of mice treated with saline, 3-CP and Branched pDHPMA-mPEG-Ppa-PROXYL at 1 day post-injection. **Fig. S12.** Biochemical tests of micetreated with saline, 3-CP and Branched pDHPMA-mPEG-Ppa-PROXYL at 1 daypost-injection.

## Data Availability

All data generated or analyzed during this study are included in this published article and its Additional file 1.

## Abbreviations

MRI: Magnetic resonance imaging
CAs: Contrast agents
mCA: Macromolecular contrast agent
3-CP: 3-Carboxy-2,2,5,5-tetramethylpyrrolidine-1-oxyl
pDHPMA: *N*-(1,3-dihydroxypropyl) methacrylamide copolymer
GSH: Glutathione
PEG: Polyethylene glycol
Ppa: Polyphthalamide
PBS: Phosphate buffer saline
TE: Echo time
TR: Repetition time
Fov: Field of view
EPR: Electronic paramagnetic resonance
SI: Signal intensity
CCK8: Cell Counting Kit-8
CLSM: Confocal laser scanning microscope
MW: Molecular weight
GFLG: Gly-Phe-Leu-Gly
PROXYL: 2,2,5,5-Tetramethylpyrrolidine-1-oxyl
TEMPO: 2,2,6,6-Tetramethylpiperidine-1-oxide
RBCs: Red blood cells
WBCs: White blood cells
HGB: Hemoglobin
HCT: Hematocrit
PLT: Platelet
MPV: Mean platelet volume
AST: Aspartate aminotransferase
ALT: Alanine aminotransferase
CREA: Creatinine
ALP: Alkaline phosphatase
GGT: Glutamyltransferase
BUN: Blood urea nitrogen
