# Supplementary material for: Amphiphilic branched polymer-nitroxides conjugate as a nanoscale agent for potential magnetic resonance imaging of multiple objects in vivo
Source: J Nanobiotechnology. 2021 Jul 9;19:205. doi: 10.1186/s12951-021-00951-z (PMC8272293; doi:10.1186/s12951-021-00951-z)
Supplement: Supplementary file 1 — Additional file 1: Scheme S1. Preparation of Branched pDHPMA-mPEG-Ppa-PROXYL. Table S1. Properties and characterizations of copolymers. Table S2. Contents of amino acids in the copolymers (wt%). Fig. S1. 1H NMR spectra of a Branched pDHPMA-SH and b Branched pDHPMA-mPEG-Ppa-PROXYL (400 MHz, d6-DMSO as solvent). Fig. S2. The EPR spectrum of Branched pDHPMA-mPEG-Ppa-PROXYL. Fig. S3. The particle size of Branched pDHPMA-mPEG-Ppa-PROXY (ca. 28 nm, DLS). Fig. S4. TEM images of Branched pDHPMA-mPEG-Ppa-PROXYL. Fig. S5. Zeta Potential of Branched pDHPMA-mPEG-Ppa-PROXYL (ca. 0 mV, DLS). Fig. S6. T1 mapping of the heart after injection of 3-CP. Fig. S7. T1 mapping ofthe aortaventralis after injection of 3-CP. Fig. S8. T1 mapping of the liver and kidney after injection 3-CP. Fig. S9. T1 mapping of the tumorsite after 3-CP injection. Fig. S10. MRI signals of Branched pDHPMA-mPEG-Ppa-PROXYL a and DTPA-Gd c. Fig. S11. Routine blood tests of mice treated with saline, 3-CP and Branched pDHPMA-mPEG-Ppa-PROXYL at 1 day post-injection. Fig. S12. Biochemical tests of micetreated with saline, 3-CP and Branched pDHPMA-mPEG-Ppa-PROXYL at 1 daypost-injection. [file 12951_2021_951_MOESM1_ESM.doc]

**Additional file 1**

*for*

**Amphiphilic branched polymer-nitroxides conjugate as a nanoscale agent for potential magnetic resonance imaging of** **multiple objects *in vivo***

Xiaoming Wang^1,3†^, Shiwei Guo^4,5†^, Zhiqian Li^1^, Qiang Luo^1^, Yan Dai^4,5^, Hu Zhang^6^, Yun Ye^4^, Qiyong Gong^1,2^ and Kui Luo^1,2*^

*1.* *Huaxi MR Research Center (HMRRC), Department of Radiology, National Clinical Research Center for Geriatrics, Frontiers Science Center for Disease-related Molecular Network, State Key Laboratory of Biotherapy, West China Hospital, Sichuan University, Chengdu, 610041, China*

*2. Functional and molecular imaging Key Laboratory of Sichuan Province, and Research Unit of Psychoradiology, Chinese Academy of Medical Sciences, Chengdu, 610041, China*

*3. Department of Radiology, Chongqing General Hospital, University of Chinese Academy of Sciences (UCAS), No.104 Pipashan Main Street, Yuzhong District, Chongqing, 400014, China*

*4. Department of Pharmacy of the Affiliated Hospital of Southwest Medical University, Southwest Medical University, Luzhou, Sichuan Province, 646000, P. R. China*

*5. Nuclear Medicine and Molecular Imaging Key Laboratory of Sichuan Province, Luzhou, 646000, P. R. China*

*6. Amgen Bioprocessing Centre, Keck Graduate Institute Claremont, CA 91711, USA*

*†Dr. Xiaoming Wang and Shiwei Guo contributed equally to this study.*

**Corresponding author, E-mail: luokui@scu.edu.cn*

**Materials and Methods**

**Materials and Analytics**

The chemical reagents used in reaction, purification and separation were of HPLC grade, and these reagents were directly used without further purification. Ppa-maleicimide, Branched pDHPMA-SH, PTE-mPEG-PROXYL were synthesized according to literature [1]. The weight-average molecular weight (MW) and polydispersity index (PDI) of the polymers were measured *via* gel permeation chromatography (GPC) on an AKTA/FPLC system (GE Healthcare). The ^1^H NMR spectrum was recorded on a Bruker 400 (400 MHz) spectrometer. The Electron Paramagnetic Resonance (EPR) spectrum was acquired using a Bruker EMX plus X-band CW spectrometer. The size and zeta potential were detected by a Zetasizer (Malvern, Worcestershire, U.K.). The target copolymer was purified and characterized by fast protein liquid chromatography (FPLC), size exclusion chromatography (SEC), ^1^H NMR analysis, amino acid analysis and dynamic light scattering (DLS) studies. Mouse breast cancer cells (4T1) and human umbilical vein endothelial cells (HUVEC) were purchased from Chinese Academy of Sciences (Shanghai) Cell Bank. The CCK-8 kit was acquired from Dojindo (Japan). Female BALB/c mice were obtained from Chengdu DaShuo Biological Technology Co., Ltd. All animal experiments were strictly in accordance with the Guidelines for Care and Use of Laboratory Animals of West China Hospital, Sichuan University and approved by the Animal Ethics Committee of China.

**Synthesis of** **Branched pDHPMA-mPEG-PROXYL**

At room temperature, Ppa-maleimide (7 mg) was added to the solution of Branched-pDHPMA-SH (100 mg, 0.17 mmol of SH) in DMF (8 mL) in a sealed tube. The above solution was stirred for 6 h at room temperature (the reaction mixture turned green). PTE-mPEG-PROXYL (719 mg, 0.28 mmol) and cat. AcOH (0.02 mL) were added to the reaction mixture with continuous stirring for 2 days, and the reaction system turned a light green transparent solution. Then 60 mL of RO-water was slowly dripped into the reaction solution, and the mixture was dialyzed against RO-water (8.0 kDa, MWCO) for 26 h. After freeze-drying, 262 mg of Branched pDHPMA-mPEG-PROXYL (light green solid) was obtained with a yield of 32%.

**Cell uptake experiment**

Confocal laser scanning microscope (CLSM) was used to observe the uptake of Branched pDHPMA-mPEG-Ppa-PROXYL by 4T1 cells. 4T1 cells were seeded on 6-well glass plates, 5 × 10^4^ cells per well, and incubated at 37 °C and 5% CO_2_ for 24 hours. Branched pDHPMA-mPEG-Ppa-PROXYL was added to each well at a concentration of 0.5 μg/mL Ppa, and the incubation was continued for 1, 2 and 6 hours, and then the cells were washed 3 times with PBS, and the nuclear dye Hoechst33342 reagent was added, followed by 20 minutes incubation. Finally, CLSM was used to acquire images to obtain fluorescence images of cell uptake materials.

***In vitro* toxicity study**

4T1 and HUVEC cells were selected to evaluate the in vitro cell toxicity of Branched pDHPMA-mPEG-Ppa-PROXYL. 4T1 and HUVEC cells were seeded into 96-well plates at a density of 1 × 10^4^ cells/well. Branched pDHPMA-mPEG-Ppa-PROXYL at different concentrations (1 mg/mL, 0.5 mg/mL, 0.25 mg/mL, 0.125 mg/mL, 0.0625 mg/mL, 0.0312 mg/mL, 0.0156 mg/mL, and 0 mg/mL) were added to the modified eagle medium (MEM medium), and the prepared media were used to replace the initial cell culture media. Cells were further incubated for 24 h and washed three times with PBS after discarding the media. The cytotoxicity evaluation kit CCK-8 (Dojindo, Japan) was added to each well. After 2 h incubation in a cell incubator, the absorbance at 450 nm was read by a multifunctional enzyme labeler (Thermo Fisher SCIENTIFIC).

**Blood compatibility test**

**RBC hemolysis test**

The anticoagulant (containing anti-citrate sodium) was added into 2 mL of fresh blood from a healthy human body. The sample was centrifuged at 1000 g for 5 min, and washed 3 times with PBS. Finally, the upper supernatant was aspirated and some red blood cells was mixed with PBS to prepare a 20% red blood cell suspension. The concentrations of Branched pDHPMA-mPEG-Ppa-PROXYL were set to 1 mg/mL, 2 mg/mL, and 5 mg/mL. PBS and deionized water were used as control groups. 1 mL of the material solution was transferred into an EP tube, followed by addition of 50 μL of red blood cell suspension. After incubation at 37 °C for 24 h, the samples were centrifuged (1000 g × 5 min). 200 μL of the supernatant was pipetted into a 96-well plate. The absorbance of the sample at 540 nm was read by a microplate reader. The above procedure was repeated three times. The percentage of hemolysis rate was calculated by a method from a previous study [2].

**Morphology and aggregation of red blood cells**

Red blood cells and the Branched pDHPMA-mPEG-Ppa-PROXYL solutions were prepared in the same manner as the above. After samples were incubated at 37 °C for 15-20 min and processed with centrifugation (1000 g × 5 min) to remove the supernatant, red blood cells were fixed with 0.5 mL of 4% paraformaldehyde for 4 h. The fixed red blood cells were re-suspended and 10-20 μL of the suspension was pipetted and evenly coated onto the bottom of a 24-well plate. The samples were successively dehydrated with 75%, 85%, 95% and 100% ethanol aqueous solution. Finally, under a constant temperature of 25-30 °C, all samples were air dried, and stereotyped and sprayed with gold. The morphologies and aggregation of cells were observed under a scanning electron microscope (SEM).

***In vivo* toxicity study**

Fifteen healthy female BALB/c mice (8-10 weeks, 20±2 g) were randomly divided into 3 groups (n = 5). Three groups of mice were injection with Branched pDHPMA-mPEG-Ppa-PROXYL and 3-CP at a dose of 0.135 mmol/kg PROXYL as well as saline (a control group) via tail vein, and all mice were sacrificed after 1 day. Main organs (heart, liver, spleen, lung, kidney) were collected, fixed with 4% paraformaldehyde solution for 48 h, and embedded in paraffin. Tissue sections were analyzed after hematoxylin-eosin staining.

Fifteen healthy female BALB/c mice (8-10 weeks, 20±2 g) were randomly divided into three groups (n = 5). Branched pDHPMA-mPEG-Ppa-PROXYL, 3-CP and saline (a control group) were injected through the tail vein at a dose of 0.135 mmol/kg PROXYL, respectively. All mice were sacrificed after 1 day. The blood samples of the mice were collected for routine blood and blood biochemical index analysis.

**Results and Discussion**

**Scheme S1** Preparation of Branched pDHPMA-mPEG-Ppa-PROXYL.

**Table S1.** Properties and characterizations of copolymers.

| compounds | MW (kDa)^a^ | PDI^a^ | Spin conc.  (mmol/g)^b^ | Ppa  content^c^ | Ionic *r_1_*  (mM^−1^ s^−1^) ^d^ |
| --- | --- | --- | --- | --- | --- |
| Branched-pDHPMA-SH [1] | 153 | 1.21 | / | / | / |
| Branched pDHPMA-mPEG-PROXYL | 160 | 1.23 | 0.059 | 0.61% | 0.50 |

^a^The MWs and PDI of the copolymers were measured *via* SEC (ÄKTA/FPLC system of GE Healthcare). ^b^ The spin conc. was expressed as the mmol/g. The Ppa content was expressed as the weight percent (wt%). ^c^ The ionic relaxivity was defined as the relaxivity per nitroxides.

**Fig. S1** ^1^H NMR spectra of **a** Branched pDHPMA-SH and **b** Branched pDHPMA-mPEG-Ppa-PROXYL (400 MHz, *d_6_*-DMSO as solvent).

**Table S2.** Contents of amino acids in the copolymers (wt%).

| Copolymer | Gly% | Phe% | Leu% | Lys% |
| --- | --- | --- | --- | --- |
| Branched pDHPMA-mPEG-PROXYL | 2.20 | 2.51 | 1.52 | 1.34 |

**Fig. S2** The EPR spectrum of Branched pDHPMA-mPEG-Ppa-PROXYL.

**Fig. S3** The particle size of Branched pDHPMA-mPEG-Ppa-PROXY (ca. 28 nm, DLS).


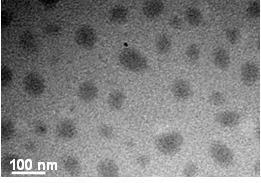


**Fig. S4** TEM images of Branched pDHPMA-mPEG-Ppa-PROXYL.

**Fig. S5** Zeta Potential of Branched pDHPMA-mPEG-Ppa-PROXYL (ca. 0 mV, DLS).


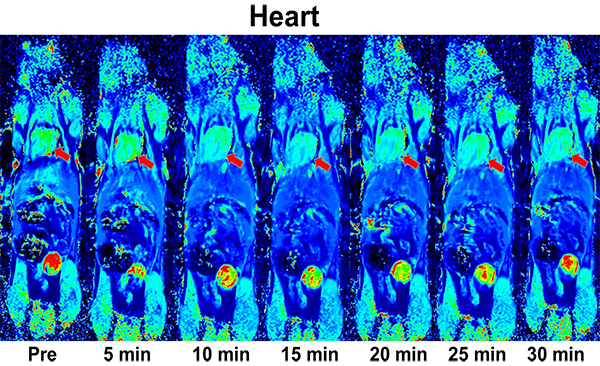


**Fig. S6** T_1_ mapping of the heart after injection of 3-CP.


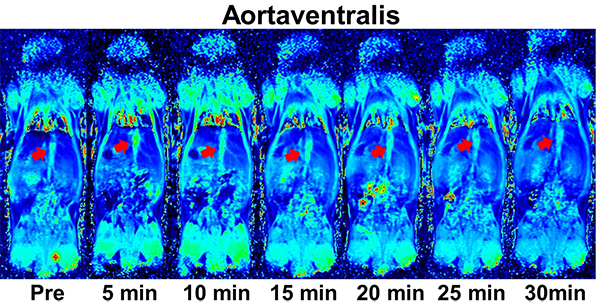


**Fig. S7** T_1_ mapping of the aortaventralis after injection of 3-CP.


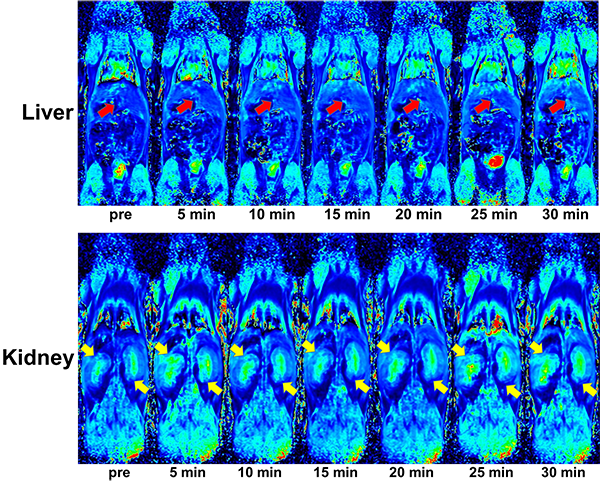


**Fig. S8** T_1_ mapping of the liver and kidney after injection 3-CP.


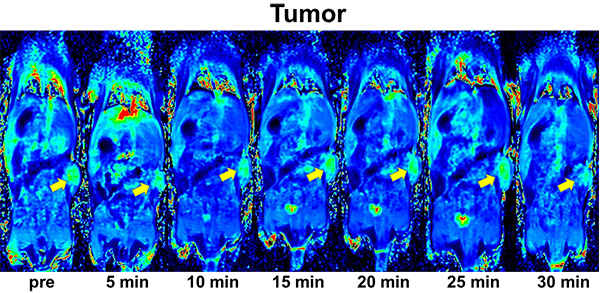


**Fig. S9** T1 mapping of the tumor site after 3-CP injection.


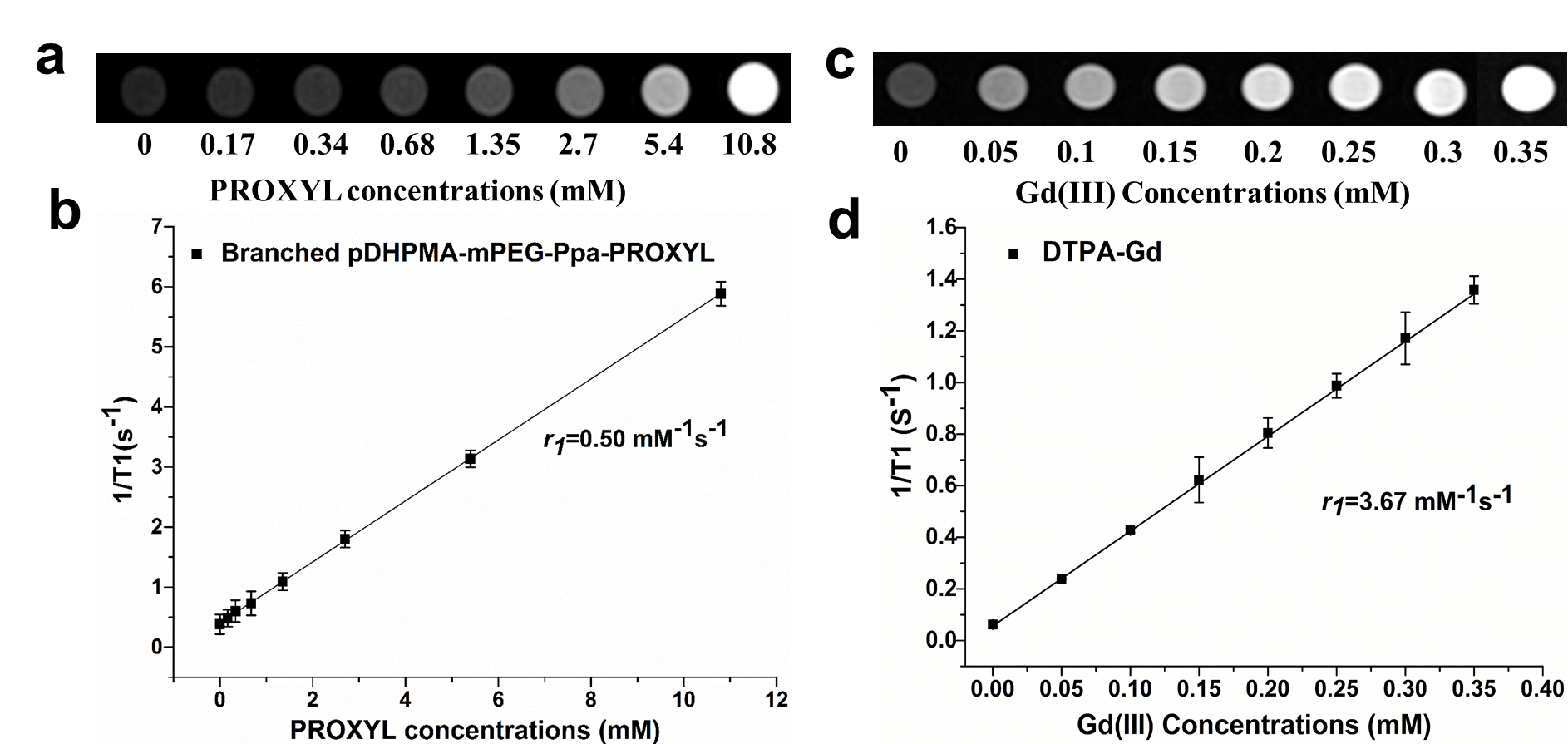


**Fig. S10** MRI signals of Branched pDHPMA-mPEG-Ppa-PROXYL **a** and DTPA-Gd **c**. In vitro longitudinal relaxivity (*r_1_*) of Branched pDHPMA-mPEG-Ppa-PROXYL **b** and DTPA-Gd **d**. A clinical Siemens 3.0 T MRI scanner was used to measure the longitudinal relaxivity (r_1_) of Branched pDHPMA-mPEG-Ppa-PROXYL and DTPA-Gd. Bright signals were seen from the DTPA-Gd samples in MRI images (Fig. S10a), and these signals were more intense than those from Branched pDHPMA-mPEG-Ppa-PROXYL (Fig. S10c). As shown in Fig. S10b, d, the *r_1_* values were calculated by plotting 1/T1 with the gradient concentration of Branched pDHPMA-mPEG-Ppa-PROXYL and DTPA-Gd. The in vitro relaxivity (*r_1_* = 3.67 mM^-1^ s^-1^) of DTPA-Gd was significantly higher than that (*r_1_* = 0.50 mM^-1^ s^-1^) of Branched pDHPMA-mPEG-Ppa-PROXYL. Although DTPA-Gd is a small molecular MRI CAs, DTPA-Gd has seven unpaired electrons, which significantly increase the energy exchange with peripheral water molecules, resulting in a significant increase in the longitudinal relaxivity. In comparison with the clinical DTPA-Gd, only one electron is associated with Branched pDHPMA-mPEG-Ppa-PROXYL, therefore, it has a small longitudinal relaxivity. Although the in vitro relaxivity of Branched pDHPMA-mPEG-Ppa-PROXYL is weaker than that of DTPA-Gd, Branched pDHPMA-mPEG-Ppa-PROXYL is a non-metallic polymer with great biosafety, which can effectively avoid the potential toxicity caused by DTPA-Gd as a metallic MR CA in clinical use. This non-metallic CA brings new opportunities for the research of new MR CAs.


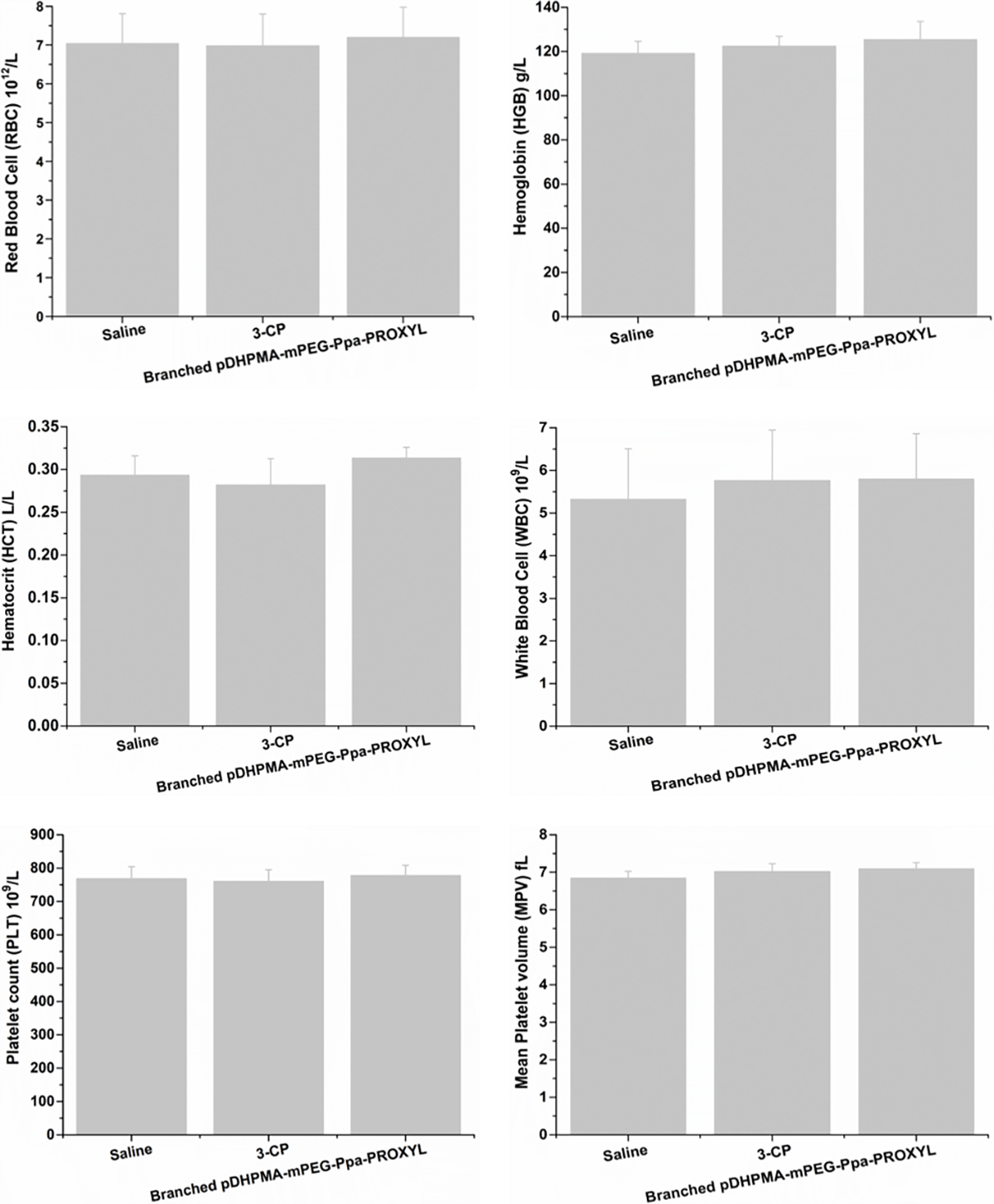


**Fig. S11** Routine blood tests of mice treated with saline, 3-CP and Branched pDHPMA-mPEG-Ppa-PROXYL at 1 day post-injection.


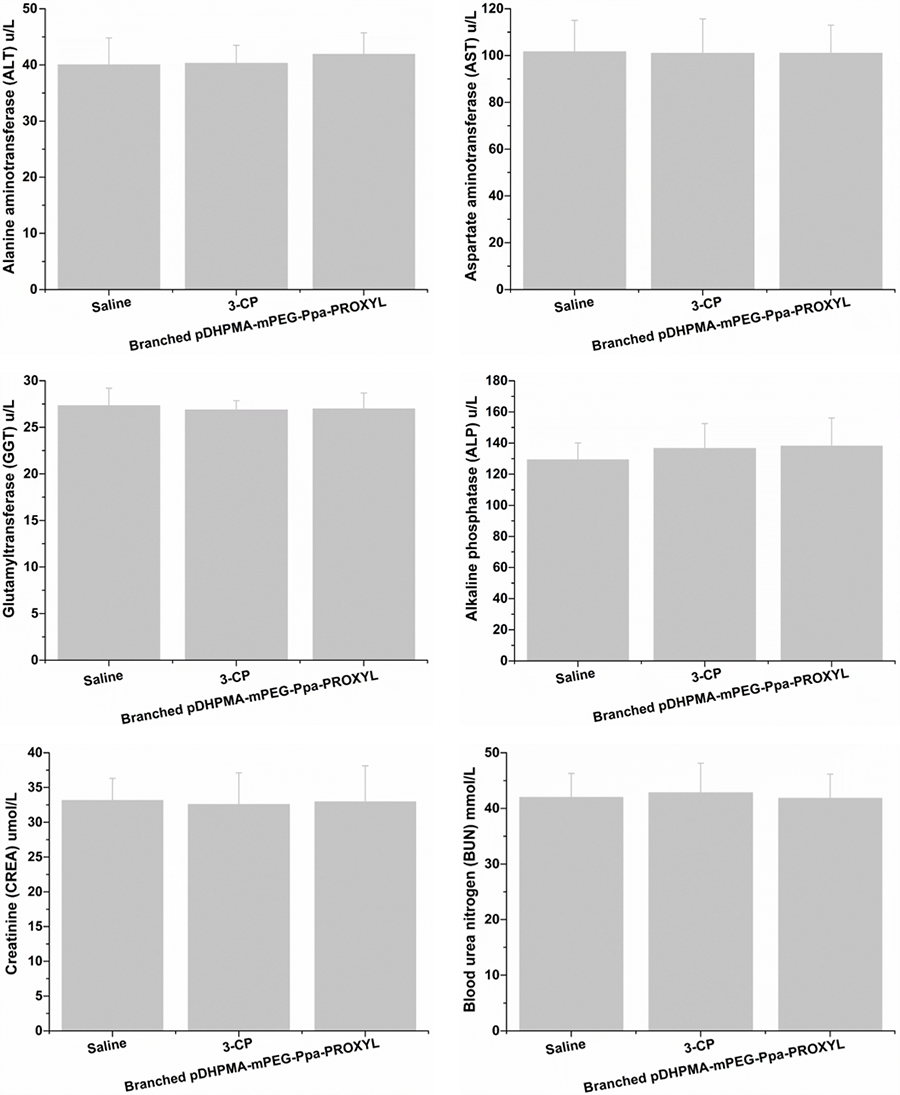


**Fig. S12** Biochemical tests of mice treated with saline, 3-CP and Branched pDHPMA-mPEG-Ppa-PROXYL at 1 day post-injection.

**Reference**

1. Wang X, Guo S, Li Z, Xiao X, Gu L, Luo Q, Zhang H, Gong Q, Luo K. Safe and potent MRI contrast agents by complexing gadolinium with enzyme/reduction dual-sensitive Branched polymers. Appl Mater Today. 2019;17:92-103.

2. Li X, Sun L, Wei X, Luo Q, Cai H, Xiao X, Zhu H and Kui L. Stimuli-responsive biodegradable and gadolinium-based poly[n-(2-hydroxypropyl) methacrylamide] copolymers: their potential as targeting and safe magnetic resonance imaging probes. J Mater Chem B. 2017;5:2763-2774.
